# Rapid and sensitive mycoplasma detection system using image-based deep learning

**DOI:** 10.1007/s10047-021-01282-4

**Published:** 2021-06-23

**Authors:** Hiroko Iseoka, Masao Sasai, Shigeru Miyagawa, Kazuhiro Takekita, Satoshi Date, Hirohito Ayame, Azusa Nishida, Sho Sanami, Takao Hayakawa, Yoshiki Sawa

**Affiliations:** 1 Department of Cardiovascular Surgery, Osaka University Graduate School of Medicine, Yamadaoka, 2-2, Suita-city, Osaka 565-0871 Japan; 2 Dai Nippon Printing Co., Ltd., Shinjuku, Tokyo Japan; 3 Osaka University Faculty of Medicine, Suita-city, Osaka Japan

**Keywords:** Mycoplasma contamination, Tissue quality control, Deep learning

## Abstract

A major concern in the clinical application of cell therapy is the manufacturing cost of cell products, which mainly depends on quality control. The mycoplasma test, an important biological test in cell therapy, takes several weeks to detect a microorganism and is extremely expensive. Furthermore, the manual detection of mycoplasma from images requires high-level expertise. We hypothesized that a mycoplasma identification program using a convolutional neural network could reduce the test time and improve sensitivity. To this end, we developed a program comprising three parts (mycoplasma detection, prediction, and cell counting) that allows users to evaluate the sample and verify infected/non-infected cells identified by the program. In experiments conducted, stained DNA images of positive and negative control using mycoplasma-infected and non-infected Vero cells, respectively, were used as training data, and the program results were compared with those of conventional methods, such as manual counting based on visual observation. The minimum detectable mycoplasma contaminations for manual counting and the proposed program were 10 and 5 CFU (colony-forming unit), respectively, and the test time for manual counting was 20 times that for the proposed program. These results suggest that the proposed system can realize a low-cost and streamlined manufacturing process for cellular products in cell-based research and clinical applications.

## Introduction

Regenerative medicine using processed cells is a promising therapy for intractable diseases, and the clinical application of such therapy is progressing [1, 2]. In the manufacturing process of cell products for regenerative medicine, biological tests such as sterility tests, endotoxin tests, and mycoplasma tests ensure the safety of biological ingredients. However, sterility and mycoplasma tests require one to several weeks because they often require a culturing process before microorganism detection. Because of such long testing times and relatively low efficiencies, these tests run the risk of increasing manufacturing costs such as the labor cost and additional costs due to delays in contamination detection, thereby making cell products expensive and hampering their practical application.

The Japanese Pharmacopoeia [3], European Pharmacopoeia [4], and US Pharmacopoeia [5] list three types of mycoplasma tests: culture method, indicator cell culture method, and nucleic acid amplification techniques (NAT). The culture method is highly reliable because it entails the direct observation of mycoplasma contamination. However, it is time-consuming, taking more than 4 weeks to produce a result. Although NAT can provide results rapidly, it does not distinguish between live and dead mycoplasma. Therefore, when NAT shows a positive result, it usually needs to be confirmed using other test methods such as the culture method, leading to a significant increase in the total test time. Although the indicator cell culture method can deliver a result in a shorter time, compared with the culture method, the examiner must be highly experienced to make a right call, because the mycoplasma contamination is checked by visual observation. In such test methods based on the observation of microscopic images, the accuracy of results may depend on the experience and expertise of observers, causing issues related to the evaluation of cell morphology in the manufacturing process of cell products and in clinical diagnosis using pathological images. As a solution, detection programs using artificial intelligence (AI) are being actively developed [6, 7]. AI can process a large number of images in a short time and may be able to detect small differences that cannot be discerned by the human eye. The development of an appropriate AI detection program is expected to reduce the test time and increase the accuracy of results. However, to the best of our knowledge, no studies using AI in mycoplasma testing have been reported.

In this study, we hypothesized that a mycoplasma detection program incorporating a convolutional neural network (CNN), which is an AI technology [8, 9], could detect, count and evaluate infection with mycoplasma, based on stained DNA images. We subsequently developed such a program and found that it yields accurate information concerning mycoplasma infections in a shorter test time with higher sensitivity compared to conventional methods.

## Methods

### Mycoplasma culture

Mycoplasma hyorhinis strains (ATCC, 17,981-TTR) were cultured for 3 days in a medium consisting of 1.75% heart infusion broth (BD, Franklin Lakes, NJ), 20% heat-inactivated horse serum (Thermo Fisher Scientific, Waltham, MA), and 10% fresh yeast extract solution (Oriental Yeast, Tokyo, Japan) under aerobic conditions (37 °C, 5% CO_2_) and subsequently freeze-preserved in a medium containing 10% glycerol. Mycoplasma arginini strains (NBRC, 111,899) were cultured for 2 days in a medium consisting of 1.5% Bacto PPLO broth (BD), 0.002% phenol red (Wako, Tokyo, Japan), 0.2% l-arginine (Tokyo chemical industry, Tokyo, Japan), 20% horse serum (Thermo Fisher Scientific), 10% fresh yeast extract solution (Oriental Yeast), 0.1% l-glutamine (Wako), and 0.1% 100X MEM vitamins (Thermo Fisher Scientific) under aerobic conditions (37 °C, 5% CO_2_) and subsequently freeze-preserved. After the cryopreserved mycoplasma were thawed and cultured, the formed colonies were counted to determine the colony-forming unit (CFU) of the cryopreserved mycoplasma.

### Cell culture

Vero, a cell line derived from African green monkey kidney, was obtained from JCRB. The cells were maintained in modified Eagle’s medium (Thermo Fisher Scientific) containing 10% fetal bovine serum (Biosera, Nuaillé, France).

### Mycoplasma tests

Indicator cells, Vero, were seeded on a cover slip in a 6-well cell culture plate in Eagle’s minimum essential medium containing 10% fetal bovine serum at 2 × 10^4^ cells/well. These cells were cultured at 37 ℃ and 5% CO_2_ for 1 day, subsequently, the medium was replaced with fresh medium, following which a positive control or negative control (Vero cells culture medium) was added and cultured at 37 °C and 5% CO_2_. As the positive control, a mycoplasma suspension prepared in 5, 10, and 100 CFU [*M. hyorhinis* (ATCC 17,981) and *M. arginini* (NBRC 111,899)] was added to the Vero cells. After 6 days, the culture medium was removed, and a methanol/acetic acid mixture (3:1) was added to each well and allowed to stand for 5 or 10 min. After removing the fixative and then completely air-drying all cover slips, 1 μg/mL bisbenzimide fluorescent staining solution (Thermo Fisher Scientific) was added to each well and allowed to stand at room temperature for 30 min. The coverslips were air-dried, mounted with a glass antifade mountant (Thermo Fisher Scientific), and examined using fluorescence microscopy at a magnification factor of 400. A test result is identified as positive if there are more than 5 cells per 1000 (0.5%) that have minute fluorescent spots that appear to surround, but are outside, the cell nucleus. The criteria for positives follow the JP XVII general information/biotechnological products 2461, B. Indicator cell culture method.

### Architecture of two-part mycoplasma detection program

**Fig. 1:**
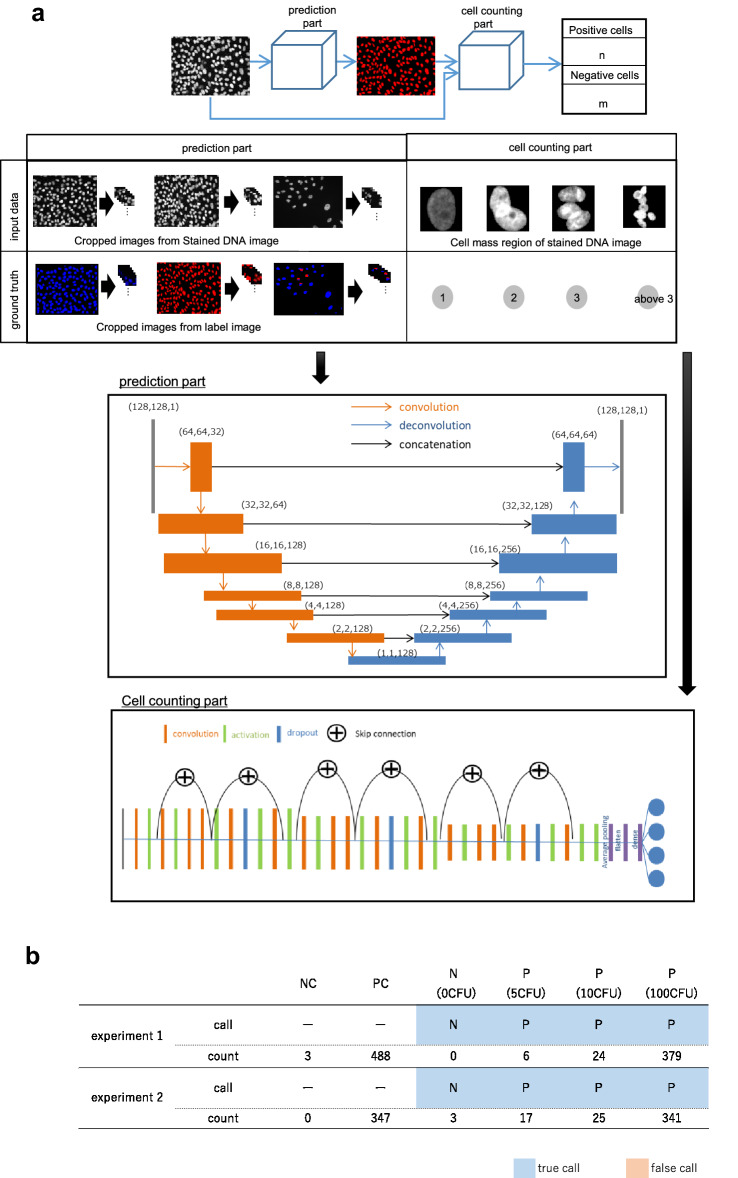
Architecture and test results of the prototype two-part program. **a** In the first part, images showing the contamination state of the indicator cells are generated from stained DNA images. 128 × 128 pixel stained DNA images that were cut out randomly from original images (1392 × 1040 pixel). In the second part, the number of cells is calculated from the images of the cell mass region obtained from the output of the first part and the stained DNA images. **b** Counted number of mycoplasma-positive cells out of 1000 cells and the call of the test. Two separate datasets were used in the experiments. *NC* negative control, *PC* positive control

Figure 1a shows an outline of the prototype program consisting of two parts. In the prediction part, regions of indicator cells are detected, and individual cells are identified as positive or negative. U-Net [10] was used for this purpose. Input data are stained DNA images, and label images show the contamination status of the indicator cells. Contaminated cells are shown in blue. Non-contaminated cells are shown in red. The input images and label images were used for training U-Net. The network outputs cell nuclei images with the contamination status of the indicator cells. The kernel size of the convolutional layer is 9 × 9 and that of the deconvolution layer is 3 × 3. The rectified linear unit is used as the activation function in both the convolutional and deconvolutional layer. The output layer has two channels: one each for contaminated and non-contaminated cells.

The number of infected and non-infected cells in the cell mass region is determined in the cell counting part. WRN (wide residual network) is used as the network in this layer [11]. The WRN used in this study was modified from the original WRN that removed dropout in skip-connection and batch normalization and used average pooling and flattening instead of GAP (global average pooling) to make the model small and fast. The WRN was used as a classification model. WRN outputs a class that represents the number of cells in the input image. The input data shown in Fig. 1a and the ground truth were used for training as label images. We created these data manually.

### Architecture of three-part mycoplasma detection program

**Fig. 2:**
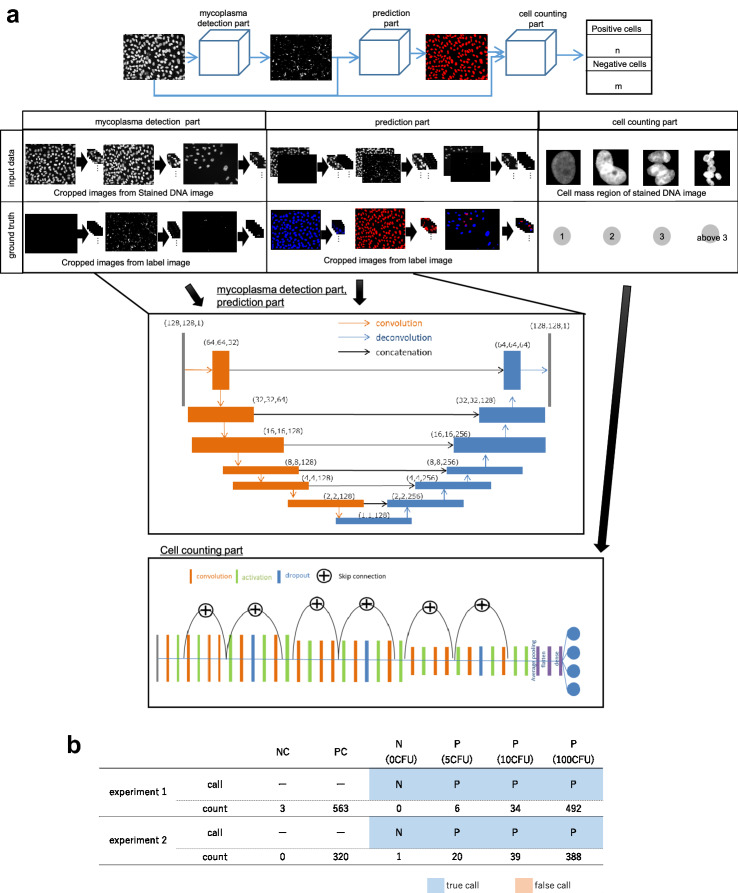
Architecture and results of the improved three-part program. **a** In the first part, the results of mycoplasma detection are generated from the stained DNA images. 128 × 128 pixel stained DNA images that were cut out randomly from original images (1392 × 1040 pixel). In the second part, images showing the contamination state of the indicator cells are generated from the stained DNA images and the output of the first part. 128 × 128 pixel stained DNA images that were cut out randomly from original images (1392 × 1040 pixel). 128 × 128 pixel mycoplasma images were cut out at the same coordinates from results of mycoplasma detection part. In the third part, the number of cells is calculated from the images of the cell mass region obtained from the output of the first part and the stained DNA images. **b** Counted number of mycoplasma-positive cells out of 1000 cells and the call of the test. Two separate datasets were used in the experiments. *NC* negative control, *PC* positive control

Figure 2a shows an outline of the program consisting of three parts. In this program, a mycoplasma detection part is added before the prediction part to visually confirm the result of mycoplasma detection. In this part, mycoplasma is detected using U-Net. The network configuration is the same as that of the prediction layer in the two-part program, and output layer has one channel. In the prediction part, the results of the mycoplasma detection part and stained DNA images are input. The network configuration is very similar to that of the mycoplasma detection part. The input layer has one channel each for the result of the mycoplasma detection part and the stained DNA images. The output layer has two channels. The cell counting part is the same as that of the two-part program. The input data shown in Fig. 2a and the ground truth were used for training as label images. We created these data manually.

### Data collection

In the prediction part of the two-part program, 128 × 128 pixel stained DNA images that were cut out randomly from original images (1392 × 1040 pixel) were input for training, and 96 × 96 pixel images showing the contamination status of the indicator cells that were cut out using the same central coordinates of stained DNA image were used as label images. In the mycoplasma detection part of the three-part program, 128 × 128 pixel stained DNA images were input, and 128 × 128 pixel images showing mycoplasma were used as label images. 128 × 128 pixel images were cut out in tiles from the stained DNA image for test. Output images were also combined in tiles. In the cell counting part, 96 × 96 pixel images of masked indicator cells were input, and the number of indicator cells was used as the label. The images of masked indicator cells were cut out from stained DNA images and areas other than those containing the indicator cells were masked to reject unwanted effects. The mask comprised binary images of indicator cells. The number of indicator cells was divided into four classes: ‘1’, ‘2’, ‘3’, and ‘above 3’.

### Performance evaluation

The constructed program was evaluated using images of Vero cells infected with a confirmed CFU of mycoplasma. The test result of the program was compared with manual counting by visual observation in terms of the call accuracy and test time. Since the mycoplasma test is not a quantitative test, the accuracy was determined by the correctness of positive/negative call and the number of mycoplasma-positive cells was not compared. Manual counting was conducted by two experts who had mastered the mycoplasma test and one novice. We used the Microsoft Cognitive Toolkit (CNTK-2.3) as a deep-learning framework, and NVIDA GeForce 2080 Ti. The program used 100th epoch trained models.

## Results

### Construction of prototype program for automatic detection and counting of mycoplasma

**Fig. 3:**
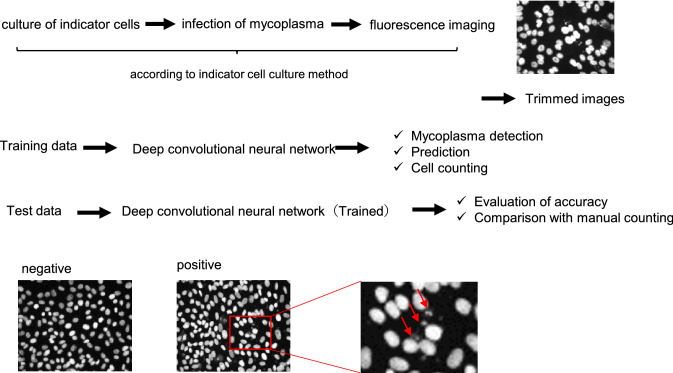
Schematic diagram of the construction of the program and evaluation. Stained DNA images of positive control (mycoplasma-infected Vero cells) and negative control (non-infected Vero cells) were used as training data. The stained DNA images were obtained using the indicator cell culture method. Representative images of positive and negative samples are shown. The red arrow indicates the observed mycoplasma

Mycoplasma tests using the indicator cell culture method consist of the following steps: culturing indicator cells with the culture supernatant of cell products, staining DNA, counting the number of mycoplasma-positive or mycoplasma-negative cells, and making an evaluation from the counted number. In this study, we developed a program for the automatic detection and counting of mycoplasma and the infection evaluation using the scheme shown in Fig. 3. The program was constructed using stained DNA images of positive control (mycoplasma-infected Vero cells) and negative control (non-infected Vero cells) as training data. To evaluate the program, test images were input to the program, and output data were verified for accuracy and compared with data from manual counting. Approximately 25,600,000 cropped images obtained from 4000 original images and 6,400,000 cropped images from 1000 original images were used as training data for positive control and negative control, respectively. Ratio of images used for training data to those used for test data was 8:2.

First, a prototype program consisting of two parts—namely, a prediction part (clarification of mycoplasma-infected and non-infected) and cell counting part (counting of mycoplasma-infected and non-infected cells)—was constructed (Fig. 1a). When images of Vero cells infected with 0, 5, 10, and 100 CFU mycoplasma were input to this program as test images, the program was able to detect as little as 5 CFU mycoplasma contamination and detect the positive sample correctly (Fig. 1b). One hundred CFU is the CFU of positive control specified by the Japanese Pharmacopoeia for the culture method, and 10 CFU is the detection limit of other mycoplasma tests, the culture method and NAT.

### Construction of improved program for automatic detection and counting of mycoplasma

**Fig. 4:**
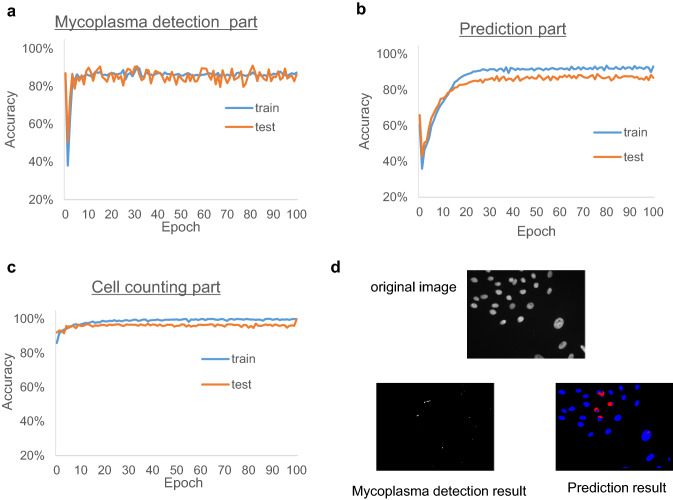
Epoch and accuracy. Learning process of the three-part program. **a–c** The accuracy of each epoch is indicated. **d** Original image, mycoplasma detection result and prediction result of 5 CFU mycoplasma

Next, to verify the distinction between infected and non-infected cells, which the prototype program showed, an improved program consisting of three parts—namely, mycoplasma detection part, prediction part, and cell counting part—was constructed based on the prototype program. As shown in Fig. 2a, the mycoplasma detection part enabled the confirmation of the distinction of the cells. When images of Vero cells infected with 0, 5, 10, and 100 CFU mycoplasma were input to this program as test images, the program was able to detect as little as 5 CFU mycoplasma contamination and detect the positive sample correctly (Fig. 2b). There were no differences between the prototype and the improved program in terms of the accuracy of negative and positive calls of the samples. Furthermore, the accuracy of the learning process in the improved program was evaluated, and the results indicate that the test accuracy in each part did not decrease and there was no over-learning (Fig. 4). Furthermore, the mycoplasma detection part and prediction part had lower accuracy than the cell counting part because different network types were used. The improved program was used for further studies.

### Comparison with manual counting

**Fig. 5:**
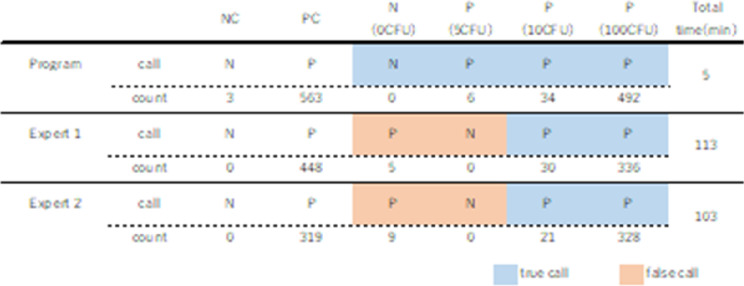
Comparison with manual counting. Counted number of mycoplasma-positive cells out of 1000 cells and the call of the test, along with the total test time

For comparison with the conventional method of manual counting by visual observation, the accuracy of call, and test time using test images (Vero cells infected with 0, 5, 10, and 100 CFU mycoplasma) were compared between the program and manual counting by two experts. While the samples infected with more than 10 CFU mycoplasma were called as positive by both manual counting and the program, samples infected with 5 CFU were called as positive only by the program (Fig. 5). In addition, samples infected with 0 CFU (non-infected samples) were falsely called as positive by manual counting, whereas the program correctly identified these as negatives. Furthermore, the total test time for manual counting was more than 100 min, while that of the program was 5 min.

**Fig. 6:**
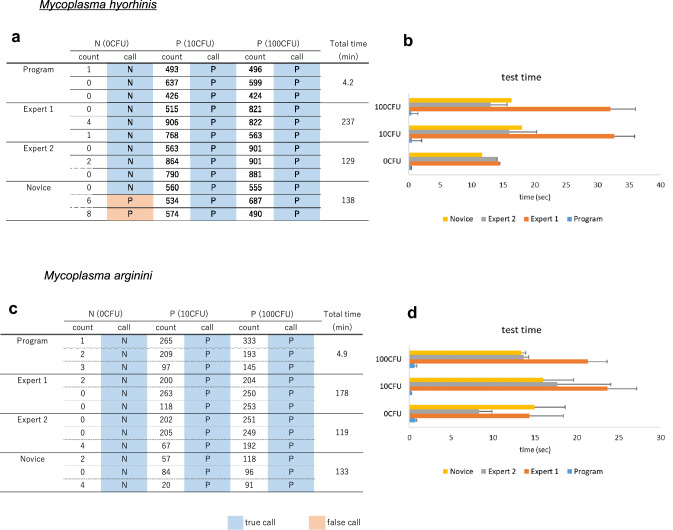
Comparison with manual counting. Counted number of mycoplasma-positive cells out of 1000 cells, the call of the test, along with the total test time (**a**), (**c**) and test time of individual samples (**b**), (**d**). Two mycoplasma species were used for the test

As two strains of mycoplasma are used as positive control in Japanese [3], European [4], and US [5] Pharmacopoeia, the program and manual counting were compared using images of samples infected with mycoplasma hyorhinis or mycoplasma arginini (0, 10, and 100 CFU). In addition, manual counting was conducted by two skilled persons (expert 1 and expert 2) and one novice. In the samples infected with mycoplasma hyorhinis, the calls of both experts and the program were correct, whereas the novice falsely identified the 0 CFU sample as positive (Fig. 6a). The total test times for expert 1, expert 2, the novice, and the program were 237 min, 129 min, 138 min, and 249 s, respectively (Fig. 6b). On the other hand, expert 1, expert 2, the novice, and the program correctly identified all the samples infected with mycoplasma arginine (Fig. 6c). The total test times for expert 1, expert 2, the novice, and the program were 178 min, 119 min, 133 min, and 293 s, respectively (Fig. 6d).

## Discussion

In this study, we constructed a mycoplasma detection program that detects mycoplasma, counts the number of mycoplasma-positive and mycoplasma-negative cells, and calls whether a sample is infected using AI technology. The program detected a mycoplasma contamination as low as 5 CFU, which was not specified in official documents as being detectable by other test methods, and correctly identified mycoplasma-positive and mycoplasma-negative samples. In contrast, the minimum detectable mycoplasma contamination with manual counting was 10 CFU. In addition, manual counting took 20 times longer to complete the test, compared with the program.

In the comparison of calls by two experts and one novice, although there were no significant differences in the test time among testers, the novice gave a false-positive call, indicating that proficiency affects calls. False-positive calls deteriorate the yield of cell products and increase product cost, while false-negative calls are not allowed in clinical cell therapy to ensure safety. Thus, call by visual observation should be avoided not only to reduce cost through the elimination of education and training of the tester, but also to ensure safety.

Deep-learning systems occasionally make decisions, based on criteria that cannot be understood by humans, and it is not possible to confirm whether the algorithm uses medically based feature quantities. In this study, using an algorithm consisting of three parts—namely, the mycoplasma detection part, prediction part, and cell counting part—the steps from interim assessment (recognition of infected and uninfected cells) to the final call can be confirmed to obtain accurate results. Since the interim assessment is visualized using U-Net [10], infected and non-infected cells can be easily confirmed from the images halfway through the evaluation the Dice coefficient was used as the loss function in U-Net in the mycoplasma detection part. Although an imbalance problem tends to occur in images such as those used in the mycoplasma test, in which the ratio of negative samples to positive samples is biased, the Dice coefficient yields better accuracy in the presence of imbalance problems than cross entropy, which is a popular loss function [11]. A WRN [12], in which the expressive power is enhanced by increasing the number of filters, was used in the cell counting part.

The techniques used in this program may also be applied to other tests that require visual evaluation by humans, such as the micronucleus test (a test for observing micronuclei appearing in the cytoplasm owing to a chromosomal abnormality) [13] and sterility test. In the case of the sterility test, image analysis using AI technology may increase sensitivity and reduce the test period by detecting minute changes that cannot be evaluated by the human eye. However, for application to other tests, it is important to prepare a considerable amount of appropriate training data for each test.

The present results indicate that the mycoplasma detection program using CNN, which detects and counts the number of mycoplasma from images, reduces the test time and increases sensitivity, suggesting that this system can effectively reduce the cost of processing cellular products.

## Data Availability

The datasets used and/or analyzed during the current study are available from the corresponding author on reasonable request.
